# Cerebellar repetitive transcranial magnetic stimulation restores pharyngeal brain activity and swallowing behaviour after disruption by a cortical virtual lesion

**DOI:** 10.1113/JP277545

**Published:** 2019-04-03

**Authors:** Ayodele Sasegbon, Masahiro Watanabe, Andre Simons, Emilia Michou, Dipesh H. Vasant, Jin Magara, Philip M. Bath, John Rothwell, Makoto Inoue, Shaheen Hamdy

**Affiliations:** ^1^ Gastrointestinal (GI) Sciences Division of Diabetes Endocrinology and Gastroenterology School of Medical Sciences University of Manchester Salford Royal Hospital (part of the Manchester Academic Health Sciences Center (MAHSC)) Salford UK; ^2^ Division of Dysphagia Rehabilitation Niigata University Graduate School of Medical and Dental Sciences Niigata Japan; ^3^ Department of Speech and Language Therapy Technological Educational Institute of Western Greece Patras Greece; ^4^ Manchester University Foundation Trust Neurogastroenterology Wythenshawe Hospital Manchester UK; ^5^ Stroke, Division of Clinical Neuroscience University of Nottingham Nottingham UK; ^6^ Sobell Department of Motor Neuroscience and Movement Disorders University College London London UK

## Abstract

**Key points:**

- Despite evidence that the human cerebellum has an important role in swallowing neurophysiology, the effects of cerebellar stimulation on swallowing in the disrupted brain have not been explored.
- In this study, for the first time, the application of cerebellar neurostimulation is characterized in a human model of disrupted swallowing (using a cortical virtual lesion).
- It is demonstrated that cerebellar stimulation can reverse the suppressed activity in the cortical swallowing system and restore swallowing function in a challenging behavioural task, suggesting the findings may have important therapeutic implications.

**Abstract:**

Repetitive transcranial magnetic stimulation (rTMS) can alter neuronal activity within the brain with therapeutic potential. Low frequency stimulation to the ‘dominant’ cortical swallowing projection induces a ‘virtual‐lesion’ transiently suppressing cortical excitability and disrupting swallowing behaviour. Here, we compared the ability of ipsi‐lesional, contra‐lesional and sham cerebellar rTMS to reverse the effects of a ‘virtual‐lesion’ in health. Two groups of healthy participants (*n* = 15/group) were intubated with pharyngeal catheters. Baseline pharyngeal motor evoked potentials (PMEPs) and swallowing performance (reaction task) were measured. Participants received 10 min of 1 Hz rTMS to the pharyngeal motor cortex which elicited the largest PMEPs to suppress cortical activity and disrupt swallowing behaviour. Over six visits, participants were randomized to receive 250 pulses of 10 Hz cerebellar rTMS to the ipsi‐lesional side, contra‐lesional side or sham while assessing PMEP amplitude or swallowing performance for an hour afterwards. Compared to sham, active cerebellar rTMS, whether administered ipsi‐lesionally (*P* = 0.011) or contra‐lesionally (*P* = 0.005), reversed the inhibitory effects of the cortical ‘virtual‐lesion’ on PMEPs and swallowing accuracy (ipsi‐lesional, *P* < 0.001, contra‐lesional, *P* < 0.001). Cerebellar rTMS was able to reverse the disruptive effects of a ‘virtual lesion’. These findings provide evidence for developing cerebellar rTMS into a treatment for post‐stroke dysphagia.

## Key points

- Despite evidence that the human cerebellum has an important role in swallowing neurophysiology, the effects of cerebellar stimulation on swallowing in the disrupted brain have not been explored.
- In this study, for the first time, the application of cerebellar neurostimulation is characterized in a human model of disrupted swallowing (using a cortical virtual lesion).
- It is demonstrated that cerebellar stimulation can reverse the suppressed activity in the cortical swallowing system and restore swallowing function in a challenging behavioural task, suggesting the findings may have important therapeutic implications.

## Introduction

Dysphagia can occur after strokes due to damage to cortical or subcortical swallowing centres. Post‐stroke dysphagia is a common complication and constitutes a significant factor in predicting stroke outcome, given its association with aspiration and pneumonia (Arnold *et al*. 2016; Cohen *et al*. 2016). Depending on the study and method of diagnosis – clinical assessment *vs*. videofluroscopy, etc. – rates of post‐stroke dysphagia range from 37 to 78% (Benjamin *et al*. 2018). Despite dysphagia being common immediately post‐stroke, some patients recover and regain their swallowing function (Smithard, 1997). One mechanism thought to drive this recovery is compensatory neuronal activity in the pharyngeal cortical representation of the undamaged hemisphere (Hamdy *et al*. 1998). Conversely, lack of recovery is associated with a failure to induce neuroplastic change (Hamdy *et al*. 1998). While the majority of the evidence available suggests the recovery of swallowing function post‐stroke is driven by this neuroadaptive process, some involvement of the damaged hemisphere in the process of recovery cannot be excluded. Evidence for this comes from interventional studies which have used high frequency excitatory repetitive transcranial magnetic stimulation (rTMS) to target cortical swallowing centres on the damaged hemisphere – in isolation or in addition to the undamaged hemisphere – in an attempt to restore normal bilateral neurophysiology (Khedr *et al*. 2009; Khedr & Abo‐Elfetoh, 2010; Park *et al*. 2017). These studies have shown that both undamaged and damaged areas may be important in the improvement of swallowing function (Khedr *et al*. 2009; Khedr & Abo‐Elfetoh, 2010; Park *et al*. 2017).

The cerebellum has a major role in the planning and organization of motor movement by modulation of the primary motor cortex through cerebello‐thalamo‐cortical connections (Suzuki *et al*. 2003; Daskalakis *et al*. 2004; Krebs, 2006). Brain imaging studies have shown activation of the cerebellum during swallowing using positron emission tomography (Hamdy *et al*. 1999) and functional magnetic resonance imaging (Mosier & Bereznaya, 2001; Suzuki *et al*. 2003). Moreover, there is evidence in the medical literature indicating swallowing can be disrupted following acute and chronic pathology affecting the cerebellum (Dailey *et al*. 1995; Izumi *et al*. 1996; Ramio‐Torrentia *et al*. 2006; Wadhwa *et al*. 2014). However, despite these studies, the physiological role of the cerebellum in the control of human swallowing remains incompletely understood.

Transcranical magnetic stimulation (TMS) is an established method for probing the mechanistic properties of human brain tissue and has been previously used to study cerebellar function (Ugawa *et al*. 1995; Daskalakis *et al*. 2004; Fierro *et al*. 2007). A seminal study by Jayasekeran *et al*. (2011) demonstrated that cerebellar TMS can induce pharyngeal motor evoked potentials (PMEPs) and therefore be used to assess cerebello‐pharyngeal physiology. Vasant *et al*. (2015) then explored the ability of differing frequencies and train durations of cerebellar repetitive transcranial magnetic stimulation (rTMS) to increase cortical PMEP amplitudes. Ten hertz cerebellar rTMS was found to have the greatest cortico‐bulbar excitatory effect over the cerebellum with frequencies above this showing comparatively reduced excitability, indicating a possible ceiling effect. Moreover, a train length of 250 pulses was found to be as effective in exciting the cerebellar pathways as longer train lengths (Vasant *et al*. 2015).

In 2007, Mistry *et al*. demonstrated that high intensity 1 Hz rTMS over the pharyngeal motor cortex was able to induce a ‘virtual lesion’ (Mistry *et al*. 2007). The effect of this ‘lesion’ was to suppress PMEP amplitudes and disrupt swallowing behaviour for up to 45 min after rTMS (Mistry *et al*. 2007). In addition, a study by Verin *et al*. (2012), using videofluoroscopy to assess swallowing function, showed that the induction of a ‘virtual lesion’ causes a similar – albeit temporary – change to swallowing, reminiscent of the changes seen after a hemispheric stroke.

In helping to better understand neuroplasticity, the ‘virtual lesion’ protocol has been developed as a model of stroke‐induced hemispheric damage (Jefferson *et al*. 2009). Indeed, studies of pharyngeal electrical stimulation (Jayasekeran *et al*. 2011) and cortical rTMS (Jefferson *et al*. 2009) have showed these two neurostimulatory techniques can reverse the suppressive PMEP and behavioural changes induced by 1 Hz lesion and have led to the development of these interventions as treatment options for dysphagic stroke. Consequently, our aim was to investigate the ability of 10 Hz cerebellar rTMS to reverse the negative effects following the creation of a 1 Hz cortical virtual lesion in the pharyngeal motor system.

## Methods

### Ethical approval

This study was granted ethical approval by Greater Manchester East Research Ethics Committees. All studies were conducted in the Gastrointestinal Laboratory at Salford Royal NHS Foundation Trust in accordance with the Code of Ethics of the World Medical Association (*Declaration of Helsinki*).

### Power calculations

Based on previous published effect size reports (Gow *et al*. 2004; Vasant *et al*. 2015), it was calculated that 12 healthy participants with complete datasets (ipsi‐lesional, contra‐lesional and sham rTMS) would be needed in order to achieve a statistical power of 80% and a *P* value of ≤0.05 assuming an effect size of 40%.

### Participant recruitment

**Figure 1:**
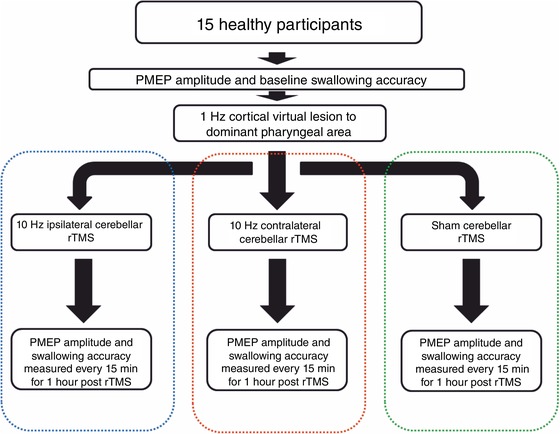
Flowchart summarizing the experimental protocol [Color figure can be viewed at wileyonlinelibrary.com]

Fifteen healthy adults were recruited for the swallowing neurophysiology and behavioural studies (Fig. 1). For the PMEP protocol 10 males and five females participated with a mean age of 30 ± 5 years. For the swallowing behaviour protocol nine males and six females participated with a mean age of 28 ± 2 years. Informed consent was obtained from all participants before initiation. Study exclusion criteria for the neurophysiology and behavioural studies included a history of epilepsy, cardiac pacemaker, swallowing problems, pregnancy, implanted metal in the head or eye, previous brain surgery or the use of medication that acts on the central nervous system.

### Outcome measures

The primary outcome measures were: (i) PMEP amplitude and (ii) swallowing accuracy. Cortical PMEP amplitude was recorded at baseline and post‐‘virtual lesion’ by delivering 10 single pulses of TMS over the dominant and non‐dominant pharyngeal motor representations, while cerebellar PMEP excitability was recorded by delivering five pulses over each cerebellar hemisphere. Swallowing accuracy was measured as the number of swallows on target out of 10 attempts using a swallowing reaction‐timing task.

### Procedures

#### Electromyography

Pharyngeal: all participants swallowed a 3.2 mm diameter Gaeltec intraluminal catheter (Gaeltec Ltd, Isle of Skye, UK) either trans‐nasally or trans‐orally depending on tolerability and preference. Each catheter comprised two circular bipolar platinum electrodes. Catheter electrodes were positioned in the middle of the pharynx 13–15 cm from the entrance to the nostrils or the lips and secured with medical tape.

Thenar: skin electrodes (H69P, Tyco Healthcare, Gosport, UK) were attached to the abductor pollicis brevis muscle on the contralateral side to the cortical hemisphere with the stronger pharyngeal representation. Thenar motor evoked potentials (TMEPs) were measured as a secondary control in order to ensure that cortical TMS and rTMS were being delivered to the pharyngeal area within specified safety limits (Wassermann, 1998).

Both pharyngeal and thenar electrodes and their corresponding earths were connected to a personal computer (Dell, Bracknell, UK) running Signal software (v4.0, Cambridge Electronic Design (CED), Cambridge, UK) via a pre‐amplifier (CED 1902). After pre‐amplification, electromyography (EMG) signals were passed through a Humbug (HumBug, Quest Scientific, North Vancouver, Canada) in order to eliminate extraneous electrical noise at 50–60 Hz. ‘Smoothed’ signals were then passed through a laboratory interface (CED micro 1401) prior to being processed by Signal software.

#### Swallowing reaction time task

Swallowing reaction times were determined using a swallowed 1.5 mm diameter manometry catheter with the sensor positioned in the same location as the pharyngeal EMG electrodes. The manometry catheter enabled pharyngeal pressures to be measured. This was attached to a custom built pressure monitoring device designed specifically to detect pharyngeal pressures (Medical Physics Department, Salford Royal Hospital Foundation Trust (SRFT), Salford, UK), operated via a purpose created swallowing timing program (Medical Physics, SRFT) run on the personal computer.

#### Magnetic resonance imaging and neuro‐navigation

A subset of the healthy participants underwent a magnetic resonance imaging (MRI) scan of their brains to confirm correct coil placement during anatomical neuro‐navigation and corroborate non‐navigated position of the coils for the remainder of the participants. Images were T1 weighted (Phillips 3T Intera‐Achieva, Amsterdam, Netherlands). Neuro‐navigation software (Brainsight 2, Rogue Research, Montreal, QC, Canada) on a computer (Apple iMac) was used to register MRI data with coil placement during the co‐registration process (Sparing *et al*. 2008; Julkunen *et al*. 2009).

#### Cortical and cerebellar single‐pulse TMS

Single‐pulse TMS was performed using a figure of eight coil with an outer diameter of 70 mm with a maximum output of 2.2 T, connected to a Magstim 200 stimulator (The Magstim Company, Whitland, UK). For cortical TMS, the coil was held in an anterior–posterior direction with the plane of the coil parallel to the scalp surface and its handle at 45° to the midsagittal line, similar to previous studies (Hamdy *et al*. 1996). For cerebellar TMS, the coil was positioned over the posterior fossa of the head, tangentially to the scalp with the handle pointing superiorly (Vasant *et al*. 2015).

#### Cortical and cerebellar rTMS

A Magstim super‐rapid stimulator (The Magstim Company) was used to deliver trains of stimuli through a figure‐of‐eight coil with a 70 mm outer diameter and a maximal output of 1.8 Tesla. To induce focal cortical suppression (the ‘virtual lesion’), 600 pulses of 1 Hz rTMS at 120% of pharyngeal resting motor threshold (RMT) were performed over the hemisphere with the largest PMEP as previously described (Mistry *et al*. 2007). High frequency stimulatory cerebellar rTMS was delivered as described by Vasant *et al*. (2015), consisting of 250 pulses of 10 Hz rTMS at 90% of thenar RMT delivered over the cerebellar hemispheres.

### Protocols

#### Protocol 1: the ability of cerebellar rTMS to reverse the suppressive PMEP effects of a 1 Hz cortical virtual lesion

Participants were asked to attend the laboratory on three occasions at least 5 days apart. All participants were asked to sit in a comfortable chair before being intubated with the EMG catheter for recording of PMEPs. Hand electrodes were then placed over the right thenar eminence.

A disposable surgical cap was positioned over the participant's head and taped down to minimize movement. The cranial vertex and inion were identified and marked on the cap.

**Figure 2:**
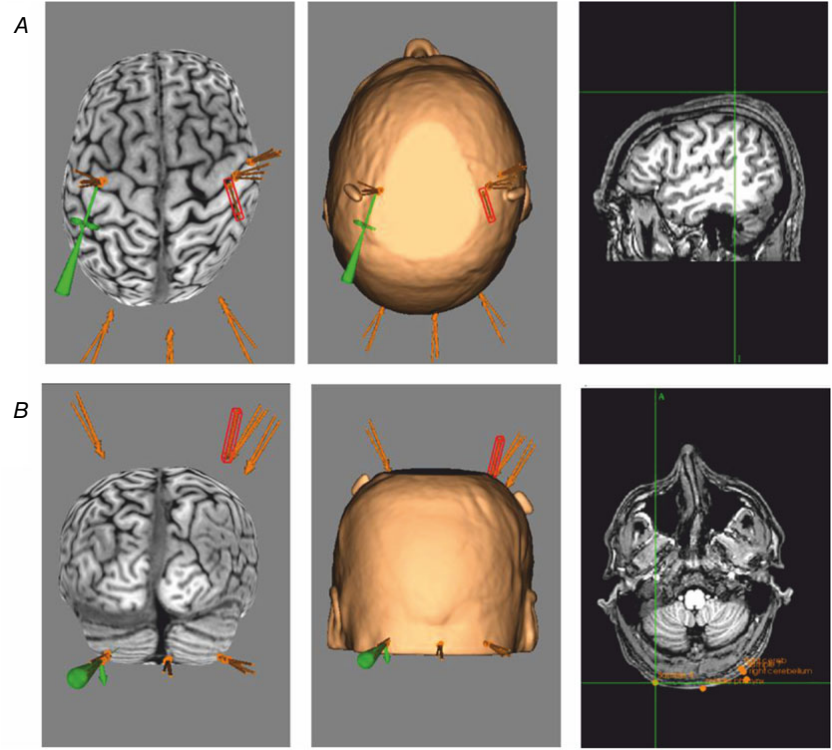
Cortical and cerebellar stimulation points from a participant's MRI using frameless functional MRI stereotaxy (Brainsight 2) [Color figure can be viewed at wileyonlinelibrary.com]

The cortical motor representations of the pharynx ‘hotspots’ and RMTs for both hemispheres were located and marked on the cap using single TMS pulses delivered through a figure of eight coil held flat against the scalp (Dailey *et al*.1995; Mosier & Bereznaya, 2001). Pharyngeal RMTs were defined as the minimum TMS stimulation intensity required to evoke cortical PMEPs >20 μV in 5 out of 10 trials. The middle of the figure of eight coil – defined as the point of intersection between both halves of the figure of eight – was the point to which focused TMS pulses were delivered. The pharyngeal cortex with the stronger responsiveness was defined as the pharyngeal cortical area with the lowest RMT and largest MEP. Cortical thenar ‘hotspots’ and RMTs – located and marked in the same way as pharyngeal ‘hotspots’ – were defined as the minimum stimulation intensity over the ‘hotspot’ required to evoke cortical TMEPs of >50 μV in 5 out of 10 trials. To identify cerebellar RMTs, the figure of eight coil was positioned over the posterior fossa of the head, flat against the scalp with its handle pointing superiorly (Vasant *et al*. 2015). The cerebellar pharyngeal ‘hotspot’ for each cerebellar hemisphere was defined as the location at which cortical PMEPs of >20 μV in 5 out of 10 trials could be evoked. MRI guided frameless stereotaxy (Brainsight 2) was used to co‐register the TMS coil position over the cerebellar hemispheres in the first two participants as a means of confirming the accuracy of the anatomical neuro‐navigation approach used for the remaining 13 healthy participants (Fig. 2).

Baseline measurements of cortical excitability, bilaterally over the pharyngeal motor representations and over the thenar motor cortex corresponding with the stronger pharyngeal cortex were recorded by delivering 10 single TMS pulses at an intensity of RMT + 20%. Baseline measurements of cerebellar excitability over both cerebellar hemispheres were recorded by delivering five single TMS pulses at RMT + 10%. During these baseline measurements, participants were asked not to swallow, cough, talk, or move their hands. Following baseline measurements of cortical and cerebellar excitability, a virtual lesion was created by delivering 1 Hz cortical rTMS at 120% of pharyngeal motor threshold for 10 min over the stronger pharyngeal motor cortex. The ‘virtual lesion’ was delivered in the same manner as performed by Mistry *et al*. (2007) and Jefferson *et al*. (2009). The ‘virtual lesion’ was immediately followed by either active or sham rTMS administered to the cerebellum. Two hundred and fifty pulses were delivered in five blocks of 50 pulses, each interspersed by 10 s, at a frequency of 10 Hz and an intensity of 90% of the thenar RMT. The three randomly allocated rTMS interventions were: (i) 10 Hz cerebellar rTMS (250 pulses) to ipsi‐lesional cerebellar hemisphere, (ii) 10 Hz cerebellar rTMS (250 pulses) to contra‐lesional cerebellar hemisphere, and (iii) sham 10 Hz cerebellar (250 pulses) to ipsi‐lesional cerebellar hemisphere.

Ten hertz cerebellar rTMS was delivered as in Vasant *et al*. (2015). The purpose of the sham arm was to provide a control by demonstrating the effect of the virtual lesion alone on cortical neuro‐electrical activity. Sham stimulation was delivered to the ipsi‐lesional cerebellar hemisphere by tilting the coil at 90° to the scalp with only the edge of one wing of the figure of eight coil in contact with the back of the head (Jefferson *et al*. 2009; Vasant *et al*. 2015).

For all study arms, cortical and cerebellar excitability measurements were recorded with single pulse TMS immediately after and every 15 min post‐intervention up to 60 min as per previous rTMS studies (Huang *et al*. 2005; Mistry *et al*. 2007).

#### Protocol 2: the ability of cerebellar rTMS to reverse the disruptive swallowing behavioural effects of a 1 Hz cortical virtual lesion

As with protocol 1, the pharyngeal EMG catheter was swallowed by each participant and thenar electrodes applied. Cortical and cerebellar pharyngeal ‘hotspots’ and RMTs were located and marked in an identical manner to protocol 1. Cortical thenar hotspots were again located and used as a secondary control.

Swallowing reaction time tasks were measured in the same manner as described by Mistry *et al*. (Mistry *et al*. 2007; Jefferson *et al*. 2009). Firstly the pharyngeal EMG catheter was removed and a manometry catheter inserted and positioned in the pharynx. This allowed swallowing pressures to be measured and timed using a reaction timing program. On opening the swallowing timing program, each participant had to undergo a period of calibration per visit. The threshold for a registered swallow was then set by hand at ∼45% of the maximal pressure of a normal swallow. Swallowing was facilitated by infusing 5 ml of water into the mouth of each participant using a sterile syringe and plastic tubing.

After baseline calibration results were obtained, participants were prompted to swallow normally 10 times followed by the instruction to swallow as fast as comfortably possible for a further 10 times. Prompts were delivered in three ways: haptically via a small electrical pulse delivered through the thenar gel electrodes (H69P, Tyco Healthcare, Gosport, UK); audibly via a clicking sound from the swallowing timing machine (Medical Physics Department, SRFT) and visually via a moving line on one of the computer screens in front of them. A swallow was prompted every 15 s. Ten challenge swallows were finally measured with all participants having to swallow accurately and hit a highlighted target time window, calculated from the normal and fast swallow average timings. The number of accurate swallows out of 10 was measured. This served as our primary behavioural outcome measure.

After baseline challenge swallows were measured, all participants had a 1 Hz ‘virtual lesion’ delivered over the stronger pharyngeal representation. Immediately after delivery of the ‘virtual lesion’, 10 Hz rTMS was delivered over the marked cerebellar areas in a similar manner to protocol 1. As before, participants then had ipsi‐lesional cerebellar rTMS, contra‐lesional celebellar rTMS and sham rTMS.

Immediately after each type of stimulation, participants had repeat measurements of their challenge swallows (10 swallows per run) at 0, 15, 30, 45 and 60 min post‐rTMS.

### Data analysis

#### Protocol 1

The latencies and peak‐to‐peak amplitudes of individual MEPs in each group of 10 traces for cortical pharyngeal and thenar areas, and in each group of five traces for cerebellar areas were averaged. Data were then compared to baseline and expressed as a percentage change from baseline.

Cortical and cerebellar MEPs were analysed separately using SPSS Statistics v. 22.0 (IBM Corp., Armonk, NY, USA). Based on previous studies (Michou *et al*. 2012; Vasant *et al*. 2015), percentage change from baseline MEP amplitudes and latencies were compared with sham using repeated‐measures analysis of variance (rmANOVA). *Post hoc* analysis using Bonferroni's correction was performed if significance was observed.

#### Protocol 2

Number of correct challenging swallows – our primary outcome – was expressed as number of swallows on target out of 10. Data were converted into percentage changes from individual baselines. Grand averages were then calculated for all participants for each intervention.

In order to determine if the study data was normally distributed, Levene's test was used to analyse all data (SPSS Statistics 22.0). As the data were not normally distributed, non‐parametric statistical methods were used. Comparisons between the effects of ipsi‐lesional, contra‐lesional and sham cerebellar rTMS effects were made using Friedman's test while comparisons between individual data points at specific times (0, 15, 30, 45 and 60 min) were made using the Wilcoxon signed rank test.

For both protocols, data are expressed as mean ± standard error of the mean unless stated otherwise. A *P* value of <0.05 was taken to indicate statistical significance.

## Results

### Cortical positioning

Using the cranial vertex as a reference point, the grand mean pharyngeal cortical locations for protocols 1 and 2 over both the right and left hemispheres were calculated. Over the right hemisphere, the mean pharyngeal location was +4.3 cm lateral and 5.8 cm anterior. Over the left hemisphere the location was −4.1 cm lateral and 5.8 cm anterior.

### Cerebellar positioning

Using the inion as a reference point, the grand mean pharyngeal cerebellar locations for protocols 1 and 2 were calculated. Over the right cerebellar hemisphere the mean pharyngeal location was +4.8 cm lateral and −2.2 cm posterior. Over the left hemisphere the location was −4.3 cm lateral and −2.6 cm posterior.

### The ability of cerebellar rTMS to reverse the suppressive PMEP effects of a 1 Hz cortical virtual lesion

There were no adverse effects associated with single‐pulse TMS or rTMS.

### Cortical and cerebellar hotspots

In all participants, TMS evoked reproducible MEPs from cortical and cerebellar hotspots. Baseline RMT, MEP amplitudes and latencies for both stronger and weaker pharyngeal areas, thenar MEPs and ipsi‐ and contra‐lesional cerebellar hemisphere PMEPs are shown in Table 1.

**Table 1:** Mean baseline cortical and cerebellar resting motor threshold (RMT), motor evoked potential (MEP) amplitudes, latencies and stimulation intensities used for each cerebellar intervention

	Ipsi‐lesional cerebellar rTMS	Contra‐lesional cerebellar rTMS	Sham
RMT (%)			
Stronger hemisphere	58 ± 2	60 ± 2	59 ± 2
Weaker hemisphere	63 ± 1	63 ± 1	64 ± 2
TMEP	40 ± 3	40 ± 3	37 ± 3
Ipsi‐lesional cerebellar PMEP	51 ± 1	49 ± 1	53 ± 1
Contra‐lesional cerebellar PMEP	51 ± 1	50 ± 1	53± 1
Amplitude (μV)			
Stronger hemisphere	105.5 ± 23.2	99.4 ± 24.0	93.3 ± 17.3
Weaker hemisphere	73.1 ± 12.6	91.9 ± 19.5	81.4 ± 14.2
TMEP	667.6 ± 145.1	490.4 ± 104.4	582.3 ± 108.3
Ipsi‐lesional cerebellar PMEP	45.4 ± 5.3	37.7 ± 5.9	37.8 ± 4.3
Contra‐lesional cerebellar PMEP	38.9 ± 3.5	52.9 ± 8.2	32.9 ± 4.7
Latency (ms)			
Stronger hemisphere	8.8 ± 0.4	8.1 ± 0.2	8.0 ± 0.2
Weaker hemisphere	9.0 ± 0.5	8.1 ± 0.2	8.2 ± 0.3
TMEP	21.4 ± 0.3	21.9 ± 0.5	21.8 ± 0.4
Ipsi‐lesional cerebellar PMEP	8.8 ± 0.2	8.3 ± 0.1	8.1 ± 0.2
Contra‐lesional cerebellar PMEP	8.8 ± 0.3	8.2 ± 0.2	8.2 ± 0.2

PMEP, pharyngeal motor evoked potential; TMEP, thenar motor evoked potential.

Of the participants, 11/15 had a stronger left cortical pharyngeal hemisphere for the ipsi‐lesional arm, 10/15 for the contra‐lesional arm and 10/15 for the sham arm, whereas the corresponding figures for the right hemisphere are 4/15, 5/15 and 5/15. One participant was observed to have a stronger cortical pharyngeal representation which switched between studies.

Of the participants, 8/15 had larger cerebellar PMEPs from the ipsilateral cerebellar hemisphere to their cortical hemisphere with the stronger pharyngeal representation, whereas 7/15 had larger PMEPs from the contralateral cerebellar hemisphere. No significant association between the stronger cortical and cerebellar hemisphere was found (Fisher's exact test, *P* = 1.00).

### Cortical PMEPs

**Figure 3:**
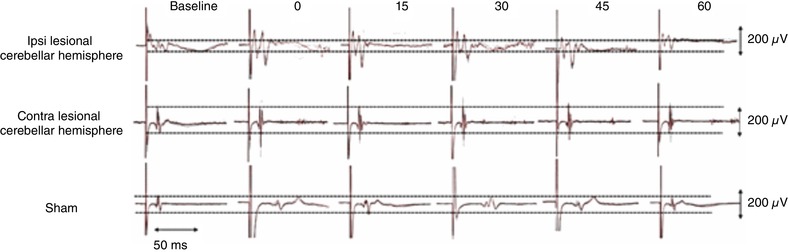
Representative PMEP traces from an individual participant at each time point Traces shown are from the virtual lesion cortical site and comprise 10 overdrawn responses before and after the 3 interventions. Horizontal dashed lines in each dataset represent peak difference of PMEP amplitude at the baseline measurement to help visualize any follow‐up amplitude changes. It can be seen that both ipsi‐ and contralateral cerebellar stimulation enhances the size of the PMEPs compared to sham stimulation. [Color figure can be viewed at wileyonlinelibrary.com]

As there were no differences in the patterns of excitability between hemispheres, PMEPs from both pharyngeal hotspots were combined and compared to sham using rmANOVA with factors of rTMS intervention (ipsi‐lesional, contra‐lesional and sham) and time. Examples of PMEPs from the stronger hemisphere of a representative participant at 0, 15, 30, 45 and 60 min for the three interventional arms are shown in Fig. 3.

Two‐way rmANOVA revealed a significant Time × Intervention interaction (*F*_2,14_ = 5.33; *P* = 0.005) and a significant effect for both ipsi‐lesional and contra‐lesional rTMS against sham (*F*_1,11_ = 11.25; *P* = 0.011, *F*_1,11_ = 14.12, *P* = 0.005).

**Figure 4:**
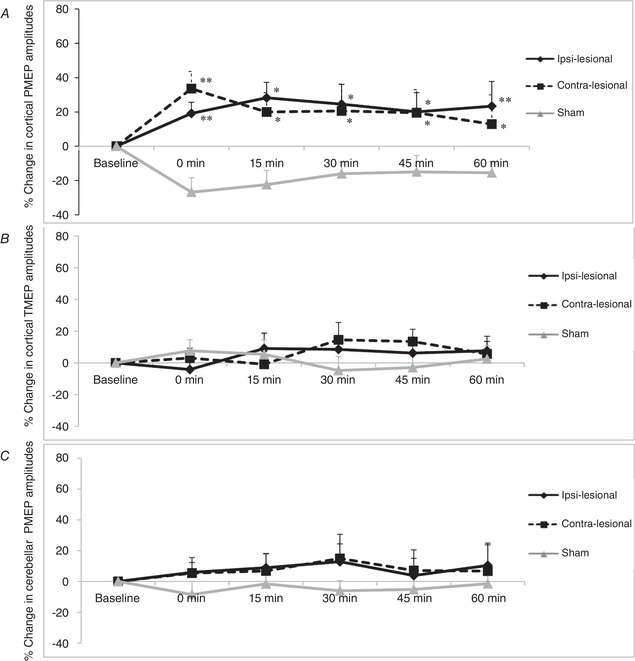
Graphs of PMEP amplitudes showing percentage changes from baseline with ipsi‐lesional, contra‐lesional and sham cerebellar rTMS following a 1 Hz cortical ‘virtual lesion’ over the pharyngeal cortical area (*A*) thenar cortical area (*B*) and cerebellar cortex (*C*) Error bars depict standard error of the mean. Note the reversal in suppressed pharyngeal cortical activity with both active cerebellar arms compared to sham. ^*^ *P* < 0.05, ^**^ *P* < 0.01.

*Post hoc* one‐way ANOVA comparing each treatment to sham confirmed that rTMS to either cerebellar hemisphere significantly increased excitability (Fig. 4), reversing the inhibition induced by the virtual lesion (ipsi‐lesional compared with Sham (*F*_1,9_ = 2.771, *P* = 0.004) contra‐lesional compared with Sham (*F*_1,9_ = 2.600, *P* = 0.001).

PMEP latencies were not affected by any intervention (Table 2).

**Table 2:** Cortical evoked response latencies from pharyngeal muscles (protocol 1)

	10 Hz rTMS on ipsi‐lesional cerebellum	10 Hz rTMS on contra‐lesional cerebellum	Sham
Cortex (combined)			
Baseline	9.0 ± 0.2	8.1± 0.1	8.1 ± 0.2
Immediately	8.9 ± 0.2	8.2 ± 0.2	8.1 ± 0.2
15 min	9.1 ± 0.2	8.3 ± 0.1	8.1 ± 0.1
30 min	9.1 ± 0.2	8.5 ± 0.2	8.2 ± 0.2
45 min	9.3 ± 0.3	8.4 ± 0.2	8.2 ± 0.2
60 min	9.1 ± 0.2	8.3 ± 0.2	8.2 ± 0.2

rmANOVA: ipsi‐lesional compared with sham: *F*_1,10_ = 1070, *P* = 0.791; contra‐lesional compared with sham: *F*_1,10_ = 2.141, *P* = 0.517.

### Cortical TMEPs

There were no differences in cortical excitability for TMEPs (ipsi‐lesional compared with sham (*F*_1,8_ = 1.823, *P* = 1.000) or contra‐lesional compared with sham (*F*_1,8_ = 1.100, *P* = 1.000) (Table 1).

### Cerebellar PMEPs

As there were no differences in the pattern of excitability between cerebellar hemispheres, PMEPs from both sides were combined and compared to sham using rmANOVA. No significant differences in cerebellar excitability were found for ipsi‐lesional (*F*_1,11_ = 2.148, *P* = 0.222) or contra‐lesional cerebellar rTMS (*F*_1,11_ = 1.871, *P* = 0.235) compared to sham (Fig. 4).

As with the cortex, PMEP cerebellar latencies were also not affected by any intervention (Table 3).

**Table 3:** Cerebellar evoked response latencies from pharyngeal muscles (protocol 1)

	10 Hz rTMS on ipsi‐lesional cerebellum	10 Hz rTMS on contra‐lesional cerebellum	Sham
Cerebellum (combined)			
Baseline	8.9 ± 0.1	8.3 ± 0.1	8.2 ± 0.1
Immediately	8.8 ± 0.2	8.3 ± 0.1	8.1 ± 0.1
15 min	8.6 ± 0.2	8.3 ± 0.1	8.1 ± 0.1
30 min	8.8 ± 0.1	8.3 ± 0.1	8.2 ± 0.1
45 min	8.8 ± 0.1	8.2 ± 0.1	8.2 ± 0.1
60 min	8.8 ± 0.1	8.3 ± 0.1	8.1 ± 0.1

rmANOVA: ipsi‐lesional compared with sham: *F*_1,8_ = 1.105, *P* = 1.000; contra‐lesional compared with sham: *F*_1,8_ = 1.468, *P* = 1.000.

### The ability of cerebellar rTMS to reverse the disruptive swallowing behavioural effects of a 1 Hz cortical virtual lesion

#### Participant demographics

As with the neurophysiological studies, all participants tolerated all study procedures well. There were no serious adverse events that occurred over the course of the study.

#### Cortical and cerebellar hotspots

Of the participants, 7/15 had stronger left cortical pharyngeal responses for the ipsi‐lesional arm, 8/15 for the contra‐lesional arm and 8/15 for the sham arm, whereas the corresponding figures for the right hemisphere were 8/15, 7/15 and 7/15. As was the case in the MEP study, one participant was observed to switch their stronger cortical pharyngeal hemisphere between studies. Baseline RMTs can be seen in Table 4.

**Table 4:** Mean baseline cortical and cerebellar resting motor thresholds (RMTs) and swallowing accuracy

	Ipsi‐lesional cerebellar rTMS	Contra‐lesional cerebellar rTMS	Sham
RMT (%)			
Stronger hemisphere	57 ± 2	55 ± 2	56 ± 2
Weaker hemisphere	63 ± 2	62 ± 2	62 ± 2
TMEP	37 ± 3	37 ± 4	38 ± 3
Number of swallows on target	4.5 ± 0.7	3.7 ± 0.6	4.8 ± 0.4

In 5/15 participants their stronger cerebellar pharyngeal representation was ipsilateral to their cortical hemisphere with the stronger pharyngeal representation, whereas 10/15 had a stronger cerebellar representation on the contralateral side. No significant association between the stronger cortical and cerebellar hemisphere was found (Fisher's exact test, *P* = 0.14).

#### Challenge swallows

**Figure 5:**
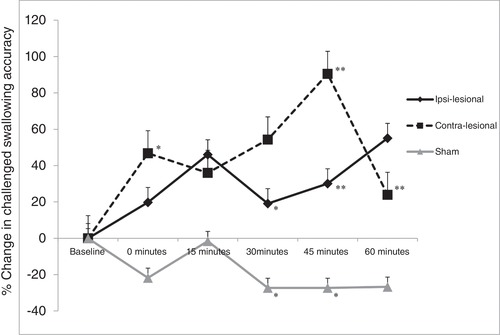
Graph of accurate challenge swallows showing percentage changes from baseline with ipsi‐lesional, contra‐lesional and sham cerebellar rTMS following a 1 Hz cortical ‘virtual lesion’ Error bars depict standard error of the mean. Note the reversal in swallowing behaviour suppressed performance with both active cerebellar arms compared to sham. ^*^ *P* < 0.05, ^**^ *P* < 0.01.

The patterns of swallowing behaviour for active cerebellar rTMS and sham cerebellar rTMS after the virtual lesion are shown in Figure 5. Visually, while there appeared a greater change in swallowing behaviour from contra‐lesional rTMS compared to ipsi‐lesional rTMS, this was not statistically significant (χ^2^ (1): *P* = 0.385).

Using Friedman's test to compare percentage changes of swallowing accuracy between interventions, both ipsi‐lesional and contra‐lesional rTMS were significantly different from sham (χ^2^ (1) = 14.92, 24.50; *P* < 0.001 and *P* < 0.001, respectively). Thereafter, time‐specific differences were assessed with the Wilcoxon test between responses from each time point. Ipsi‐lesional rTMS was better than sham at 30, 45 and 60 min (*Z* = −2.101, −2.606 and −2.445; *P* = 0.035, 0.007 and 0.012, respectively). Contra‐lesional rTMS was better than sham, baseline and ipsi‐lesional rTMS at 45 min (*Z* = −3.124, −2.791 and −2.199; *P* = 0.001, 0.003 and 0.025, respectively) and sham at 0 and 60 min (*Z* = −2.101 and −2.637; *P* = 0.036 and 0.006, respectively). By comparison, sham was worse than baseline at 30 and 45 min (*Z* = −2.075 and −2.553; *P* = 0.037 and 0.007, respectively).

## Discussion

Our results show that both ipsi‐lesional and contra‐lesional cerebellar rTMS were able to reverse the PMEP suppression and disrupted swallowing behavioural effects of a ‘virtual lesion’ to the pharyngeal motor cortex. However, there was no clear physiological difference between (active) cerebellar rTMS (ipsi‐lesional and contra‐lesional), implying a non‐selective cerebellar facilitation of the cortex for the swallowing neural network. These findings provide supportive evidence for this being a potential treatment for dysphagic stroke, thus meriting further discussion.

Our results demonstrate it is possible to reverse an experimentally induced cortical lesion by administering rTMS to either cerebellar hemisphere. There were significant differences between the active treatment arms (ipsi‐lesional and contra‐lesional) and sham but there was no difference between the two active arms. This suggests that cerebellar rTMS is equally good at reversing the disruptive effects of the lesion regardless of which side it is administered. Efferent projections from both cerebellar hemispheres primarily send impulses to motor areas in the respective contralateral cerebral cortices via dentate nuclei whose axons exit the cerebellum through the superior cerebellar peduncle before synapsing in the thalamus (Krebs, 2006; Moore & Dalley, 2006; Roostaei *et al*. 2014). These pathways provide an explanation as to the neurophysiological mechanism for contralateral cerebellar rTMS to reverse the effects of a ‘virtual lesion’. However, for ipsilateral cerebellar rTMS, the situation is less clear cut. The fastigial nuclei in each cerebellar hemisphere send projections to various locations in the medulla including the nucleus ambiguus (Zhang *et al*. 2016). The nucleus ambiguus and the nucleus tractus solitarii together form the central pattern generator (CPG) which is responsible for the subcortical control of swallowing (Bostan *et al*. 2013; Ludlow, 2015; Sasegbon & Hamdy, 2017). As mentioned above, the CPG is bilaterally connected to higher swallowing centres in the cortex (Sasegbon & Hamdy, 2017) with interneuronal communication between CPG nuclei. This lower circuitry connectivity provides a potential explanation for the reversal effect seen with ipsilateral cerebellar rTMS. Alternatively ipsi‐lesional cerebellar stimulation may indirectly affect the lesioned hemisphere via its excitatory effects on the contralateral hemisphere. Subsequent communication through transcallosal or inter‐hemispheric motor–motor connections would then explain any ipsilateral effect. Lastly, it may be that as well as stimulating the cerebellar cortex, rTMS also stimulates deeper cerebellar motor nuclei bilaterally, hence explaining its ipsilateral effect. The cerebellum in its interactions with deep cerebellar and vestibular nuclei – via axons from the Purkinje cell layer – is primarily an inhibitory organ. It is through selective inhibition of brainstem and cortical inputs that coordinated motor activity and learning is able to occur (Roostaei *et al*. 2014). Cerebellar rTMS has the potential to modulate inhibitory responses and in so doing affect brainstem and cortical function. This suggests the possibility of cerebellar rTMS activating differentially excitatory and inhibitory neuronal pathways when applied at different frequencies or over different cerebellar regions. A possible example of this are corticopontine neurones which project from the cerebral cortex to the cerebellar hemispheres through the middle cerebellar peduncle (Bostan *et al*. 2013). These neurones are different from those in the cerebellar vermis (Zhang *et al*. 2016). This implies that cerebellar rTMS applied over the midline may have a subtlety different modulatory effect on the cerebral cortex than hemispheric stimulation.

Our results confirm findings from earlier ‘virtual lesion’ studies (Mistry *et al*. 2007; Jefferson *et al*. 2009) where the sham arm was associated with reduced PMEP amplitudes, indicating that the virtual lesion is a robust model for cortical inhibition. The reduction in cortical activity following a ‘virtual lesion’ can be seen as being analogous to the reduction in cortical activity observed after a stroke (Hamdy *et al*. 1998). While there are no direct comparisons of cerebellar rTMS being applied to reverse the disruptive MEP effects of a virtual lesion, Jefferson *et al*. did find that contra‐lesional cortical rTMS was able to restore the PMEP and behavioural effects of a cortical ‘virtual lesion’ (Jefferson *et al*. 2009). This implies that cerebellar rTMS has at least similar efficacy in reversing any suppressed post‐‘virtual lesion’ MEP amplitudes and resolving negative swallowing behavioural effects. Moreover, Michou *et al*. (2014) investigated the ability of cortical rTMS, pharyngeal electrical stimulation and paired associated stimulation (PAS) to improve cortical activity over the pharyngeal motor cortex in eighteen patients with post‐stroke dysphagia. They found increased coticobulbar excitability over the unaffected hemisphere after all neurostimulatory techniques (Michou *et al*. 2014). Pisegna *et al*. (2016) performed a meta‐analysis of the effects of two different non‐invasive neurostimulatory methods – cortical rTMS and transcranial direct current stimulation – on post‐stroke dysphagia. RTMS was found to have a significant moderate beneficial effect on post‐stroke dysphagia (Pisegna *et al*. 2016). Furthermore, Hamdy *et al*. (1998) showed recovery of dysphagia post‐stroke is associated with an increase in activity over the undamaged hemisphere. This in combination with study of Pisegna *et al*. corroborates the focus on undamaged pathways where the application of neurostimulation to the unaffected hemisphere seems to have greater beneficial effect on dysphagia compared to the affected hemisphere.

Behaviourally, we were able to show that swallowing accuracy, as measured using a challenging swallowing reaction task, improved with active cerebellar stimulation compared to both baseline and sham cerebellar rTMS. Sham cerebellar rTMS resulted in a consistent reduction in swallowing accuracy following the virtual lesion. This is in agreement with the findings of other researchers (Mistry *et al*. 2007; Jefferson *et al*. 2009; Verin *et al*. 2012) all of whom found reduced swallowing accuracy after 1 Hz cortical suppression. By contrast both ipsi‐lesional and contra‐lesional cerebellar rTMS showed a significant improvement in swallowing accuracy. It is reasonable to suppose that cerebellar rTMS may have a dual role. Firstly, it directly reverses the effects of the cortical suppression – as has been demonstrated in previous studies (Verin *et al*. 2012) – through efferent connections between the cerebellum and the cortex (Michou *et al*. 2012). Secondly any increase of intrinsic cerebellar activity provoked by rTMS may increase its role in modulating fine motor activity (Michou *et al*. 2012), thereby increasing the accuracy of executed movements. As the effects of a cortical ‘virtual lesion’ on swallowing behaviour are similar to the swallowing seen in post‐stroke dysphagia (Verin *et al*. 2012), it can be argued that these findings support translation of neurocerebellar stimulation into therapy after dysphagic stroke without the need to choose the side for stimulation.

From a clinical point of view, cerebellar rTMS has the potential to be a novel treatment for neurogenic dysphagia. However, because there have been very few published studies in this area, it is not currently known what parameters are needed for the optimal stimulatory regime, so as to provoke the greatest beneficial neuroplastic and rehabilitative effect. Clinical trials involving the more well studied technique of cortical rTMS have studied a number of stimulatory regimes ranging from isolated single applications of rTMS to multiple applications over time periods of up to 2 weeks (Lim *et al*. 2014; Michou *et al*. 2014; Momosaki *et al*. 2014). As yet no consensus has been reached. Regarding cerebellar rTMS, similar trials are needed which investigate the initial and the dose‐related effect of this technique on patients with post‐stroke dysphagia. Stimulation of the cerebellum has several potential advantages over cortical rTMS. Firstly, cerebellar rTMS may improve post‐stroke dysphagia in patients with posterior fossa strokes. Cortical rTMS and peripheral neurostimulation techniques have offered little benefit in this patient group. Secondly the human cerebellum is much easier to locate and stimulate using anatomical landmarks than other pharyngeal regions of the brain. This means cerebellar rTMS may require less training or precision to administer. This may have a direct impact on the availability of this technique for patients with neurogenic dysphagia. Finally, rTMS over the cerebellum has a lower risk of serious adverse effects such as seizures (Rossi *et al*. 2009).

Despite these novel findings, our study does have some limitations. First, the study analysed the neurophysiology and behaviour in two sets of overlapping participants. Further studies, using greater numbers of participants should strengthen these data and may result in the emergence of significance between active treatment arms (ipsi‐lesional and contra‐lesional). Second, all participants in this study were relatively young (average age 28.3 years). This is in contrast with the much higher age of stroke patients (Benjamin *et al*. 2018; Office for National Statistics 2016*a*,*b*). Thirdly, while the behavioural task of swallowing performance was a robust experimental method to study swallowing function, we did not apply more detailed imaging such as fluoroscopy or endoscopy to our participants, which might be considered a more comprehensive swallowing evaluation, albeit being more invasive and exposing participants to ionizing radiation. Fourthly, within the parameters of this study, we cannot be completely certain that all the effects observed are solely due to cerebellar modulation. However, several factors suggest that that is indeed the case. Two previous studies by our group have shown that measurable cerebellar PMEPs –morphologically similar to cortically evoked PMEPs – can be evoked using single pulse TMS over the cerebellar hemispheres and more weakly from the cerebellar vermis (Jayasekeran *et al*. 2011; Vasant *et al*. 2015). In addition a study by Ugawa *et al*. found that a double cone coil – as opposed to a figure of eight coil – was needed to reliably evoke brainstem motor responses (Ugawa *et al*. 1995). Finally, performing sham stimulation with a tilted figure of eight TMS coil in contact with the scalp is not a perfect replica of active rTMS stimulation. It approximates the experience of receiving rTMS by replicating the pressure on the scalp and the sound of the electromagnetic pulses as they travel through the coil. However, it cannot replicate the sensation of TMS pulses traveling through the scalp. This method, although imperfect, has been used in multiple published studies in this area (Jayasekeran *et al*. 2011; Vasant *et al*. 2015), is well tolerated, and appears to provide a close to placebo effect.

In conclusion, we found both pharyngeal brain excitability and swallowing accuracy were significantly better with cerebellar rTMS compared to sham following a ‘virtual lesion’. As such, 10 Hz cerebellar rTMS is able to restore the disruptive neurophysiological and behavioural responses to cortical lesions suggesting it may be useful in dysphagic stroke.

## Additional information

### Competing interests

None declared.

### Author contributions

A. S. performed the studies in conjunction with M.W., J.M., A.S. and E.M. A.S. wrote the paper and analysed the data along with M.W. and E.M. D.V., J.R., P.B. and M.I. helped with conceptualization of the study, data interpretation and critical revision of the manuscript. S.H., J.R. and P.B. obtained funding. S.H. conceptualized and supervised the study, and helped with data interpretation and writing of the manuscript. All authors had access to the study data and reviewed the final manuscript. All authors have read and approved the final version of this manuscript and agree to be accountable for all aspects of the work in ensuring that questions related to the accuracy or integrity of any part of the work are appropriately investigated and resolved. All persons designated as authors qualify for authorship, and all those who qualify for authorship are listed.

### Funding

Funding for this study was provided by the Medical Research Council (MRC). MR/P006183/1. The authors would also like to thank the Strategic Young Researcher Overseas Visits Program for Accelerating Brain Circulation (S2504) from the Japan Society for the Promotion of Science. P.M.B. is Stroke Association Professor of Stroke Medicine and is a NIHR Senior Investigator.
